# Supplementary material for: Gold endowments of porphyry deposits controlled by precipitation efficiency
Source: Nat Commun. 2020 Jan 14;11:248. doi: 10.1038/s41467-019-14113-1 (PMC6959242; doi:10.1038/s41467-019-14113-1)
Supplement: Supplementary file 2 — Supplementary Information [file 41467_2019_14113_MOESM2_ESM.pdf]

Supplementary Information

Gold endowments of porphyry deposits controlled by precipitation efficiency

Massimo Chiaradia*

Department of Earth Sciences, University of Geneva
Rue des Maraîchers 13, 1205 Geneva, Switzerland

**Corresponding author: Tel.: +41 22 379 66 34; Fax: +41 22 379 32 10; e-mail: Massimo.Chiaradia@unige.ch*

Supplementary Note 1

Details of calculations of duration of ore processes

Bajo de la Alumbrera (Buret et al. 2016)[1]
Duration of ore process is estimated from the age difference, including propagation of uncertainty (0.021±0.028 Ma), between zircon U-Pb age of pre-syn mineralization porphyry P2 (7.1102±0.0093/0.012) and molybdenite Re-Os age of 7.089± 0.020/0.025 Ma. If the age difference is taken between U-Pb zircon age of P2 and U-Pb zircon age of post-mineralization LP3 (7.0963±0.0085/0.012) then the duration of the ore process is 0.014±0.014 Ma.

Batu Hijau (Garwin 2002)[2]
Inferred by the author from $^{40}Ar/^{39}Ar$ dating: 80±80 ka.

Bayugo (Braxton et al., 2012)[3]
Pre-mineralization intrusions are dated at 2.31±0.1 and 2.23±0.1 Ma by U-Pb in zircon. Latest late-ore porphyryies are dated by U-Pb in zircon at 2.19±0.16 Ma at Bayguo (duration of ore process is 0.120±0.189 or 0.040±0.189 Ma) and at 2.09±0.2 Ma at Boyongan (duration of ore process is 0.22 ± 0.189 or 0.14±0.189 Ma).

Bingham (von Quadt et al., 2011)[4]
Duration of ore process is 0.32 Ma as inferred by the authors based on U-Pb zircon ages of 3 porphyry intrusions associated with two Cu-Au mineralization pulses.

Butte (Dilles et al., 2004)[5]
Duration of ore process is ~1.5 Ma as inferred by the authors based on combined U-Pb and Re-Os ages.

Chaucha (Schütte et al., 2012)[6]
Porphyry intrusion dated at 9.79±0.03 Ma by U-Pb in zircon plus 2 Re-Os molybdenite ages at 9.91 ± 0.04 (Naranjos) and 9.54± 0.05 (Gur-Gur): the 2 latter give a duration of ore process of 0.37±0.064.

Chuquicamata (Barra et al. 2013; Ballard et al., 2001)[7,8]
Ore duration is based on age range of 6 Chuquicamata molybdenite samples dated by Re-Os (32.9±0.2 to 31.7±0.2 = 1.2±0.28 Ma) (Barra et al., 2013).
Ore duration based on U-Pb zircon ages of two distinct porphyry intrusions is 1.3±0.36 Ma (Ballard et al., 2001).

Coroccohuyaco (Chelle-Michou et al., 2015)[9]
Duration of ore process inferred by the authors is 0.1 Ma, based on combined U-Pb on hydrothermal skarn titanite and Re-Os in moly (35.7-35.6 Ma).

El Abra (Correa et al., 2016)[10]
Duration of ore process is 0.16±0.26 Ma based on 3 molybdenite Re-Os ages spanning from 36.34±0.18 to 36.18±0.18 Ma.

El Salvador (Zimmermann et al., 2014)[11]
Duration of ore process is 0.6 Ma based on 15 Re-Os molybdenite ages ranging from 41.8 to 41.2 Ma.

El Teniente (Maksaev et al. 2004; Cannell et al., 2005)[12,13]
Duration of ore process is 1.88± 0.04 Ma based on 7 molybdenite Re-Os ages from Maksaev et al. (2004) ranging between 6.30±0.03 and 4.42±0.02 Ma.
Duration of ore process is 1.2 Ma based on 14 Re-Os moly ages from Cannel et al. (2005) ranging from 5.89 to 4.70.

Elatsite (von Quadt et al., 2002)[14]
Duration of ore process is 0.26±0.42 Ma, bracketed by U-Pb zircon ages of the pre- to syn-ore quartz monzodiorites of the big dyke(HOR1390, unit 1) (92.1±0.3 Ma) and zircons from the latest ore-forming granodiorite porphyry dike (LF 025, unit 2) at 91.84 ± 0.3 Ma.

Far Southeast-Lepanto (Hedenquist et al., 1998)[15]
Duration of ore process inferred by authors is 0.3 Ma with peak activity ≤0.1 Ma, based on $^{40}Ar/^{39}Ar$ dating of hydrothermal minerals.

Golpu-Wafi (Rinne et al., 2018)[16]
Duration of ore process inferred by the authors is 120-220 ka based on a combination of U-Pb zircon of porphyry intrusions, Re-Os molybdenite and $^{40}Ar/^{39}Ar$ ages (hydrothermal biotite, alunite). Most ore is inferred to be formed within 55-147 ka.

Grasberg (Wafforn, 2017; Pollard et al., 2005)[17,18]
Duration of the Grasberg PCD is 0.13±0.64 Ma, based on U-Pb zircon of porphyry intrusions bracketing the mineralization from 3.22±0.04 to 3.09±0.05 Ma. If the younger ages of the Big Gossan skarn (U-Pb ages of garnet dated between 2.87±0.07 and 2.72±0.04 Ma) are included the total duration extends to 0.5±0.057 Ma.
Pollard et al. (2005) infer a duration between 0.3 and 0.5 Ma from a combination of U-Pb zircon, Re-Os molybdenite and $^{40}Ar/^{39}Ar$ ages of alteration minerals.

Junin (Schütte et al., 2012) [6]
Duration of ore process is 0.5±0.036 Ma, based on 2 molybdenite Re-Os ages (6.63±0.03 and 6.13±0.02).

Kisladag (Baker et al., 2016; Paolillo, 2018)[19,20]
Duration of ore process is <0.4 Ma[19] and ≤0.2 Ma[20], based on combined U-Pb on zircons of porphyry intrusions and Re-Os of several molybdenite samples.

Los Pelambres (Stein et al., 2002)[21]
Duration of ore process is ~1 Ma inferred by authors based on Re-Os dating of molybdenite.

Ok Tedi (Large et al., 2018)[22]
Duration of ore process is 0.075±0.030 Ma, based on the youngest pre-ore porphyry (U-Pb zircon) and the youngest syn-ore porphyry dated at 1.262±0.02 and 1.187±0.022 Ma, respectively.

Pebble (Lang et al., 2013)[23]
Duration of ore process is 0.5±0.72 Ma from 5 Re-Os molybdenite ages measured at University of Alberta and reported by Lang et al. (2013).

Qulong (Li et al., 2017)[24]
Duration of ore process is 0.35 Ma based on Re-Os on molybdenite and cross-cutting relationships, bracketed by ages ranging from 16.10±0.06 to 15.88±0.06 Ma (age difference = 0.220±0.085 Ma).

Reko Diq (Razique et al., 2014)[25]
Duration of ore process is 0.49±0.194 Ma, based on U-Pb zircon dating of H15 and H14 main ore stages porphyries bracketed by the oldest H15 (12.49±0.11 Ma) and the youngest (late mineral) H14 (12.00±0.16 Ma). Using only main ore stage porphyries H15 12.49±0.11 and H14 12.05±0.15 Ma, the duration of ore process is 0.44±0.19 Ma.

Rio Blanco (Deckart et al., 2013)[26]
Duration of ore process is 1.44±0.036 Ma, based on 10 Re-Os molybdenite ages with range from 5.94±0.03 to 4.50±0.02 Ma.

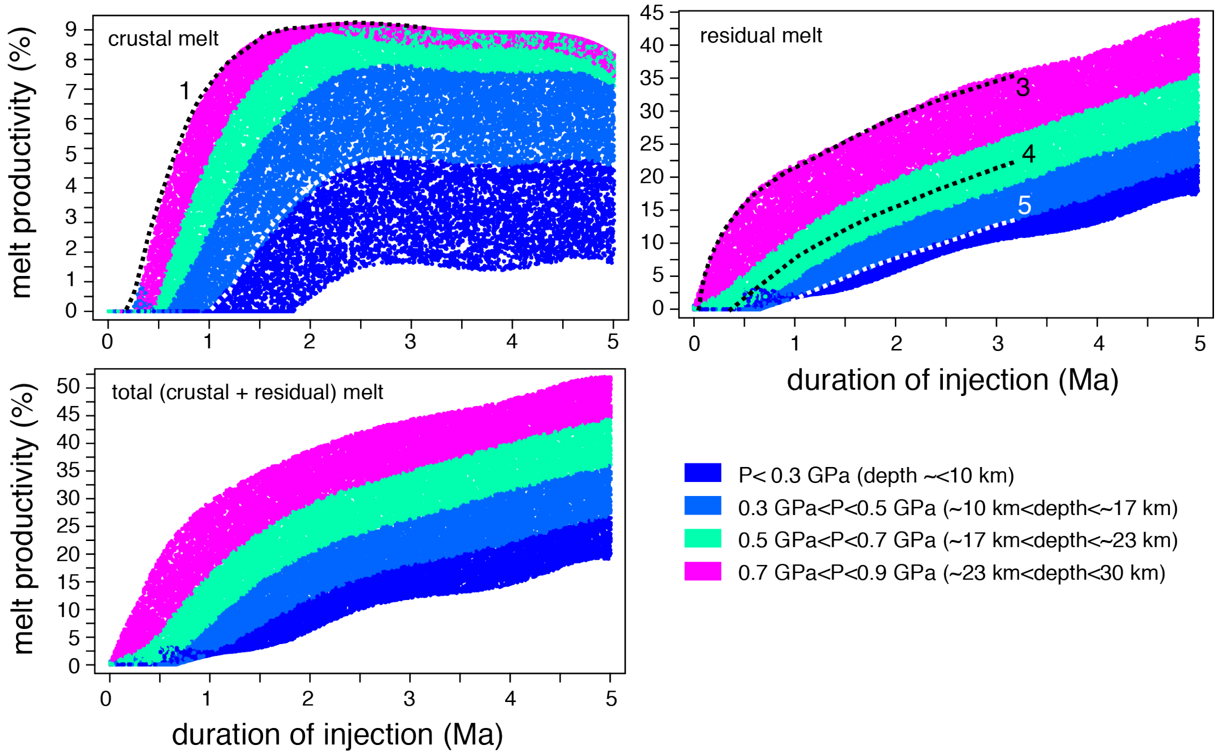

Supplementary Figure 1: *Results of the parameterization of melt productivities for crustal and residual melts from the model of Annen et al. (2006)[27] and their Monte Carlo simulations. Dashed curves represent the curves of the model of Annen et al. (2006)[27] and the colored dots represent the results of parameterization and ~100000 Monte Carlo simulations. Dashed curves labels: 1 is the crustal melt productivity from the model of Annen et al. (2006)[27] at depth of 30 km from partial melting of amphibolitic crust; 2 is the crustal melt productivity from the model of Annen et al. (2006)[27] at depth of 10 km from partial melting of graywacke crust; 3, 4, 5 indicate residual melt productivities from the model of Annen et al. (2006)[27] for basalt fractionation at depths of 30 km, 20 and 10 km respectively. The results of the model of Annen et al. (2006)[27] have been extrapolated to higher durations of basaltic injections (5 Ma) and to slightly lower pressures (0.15 GPa). This Figure is from the Supplementary Information of ref. 28.*

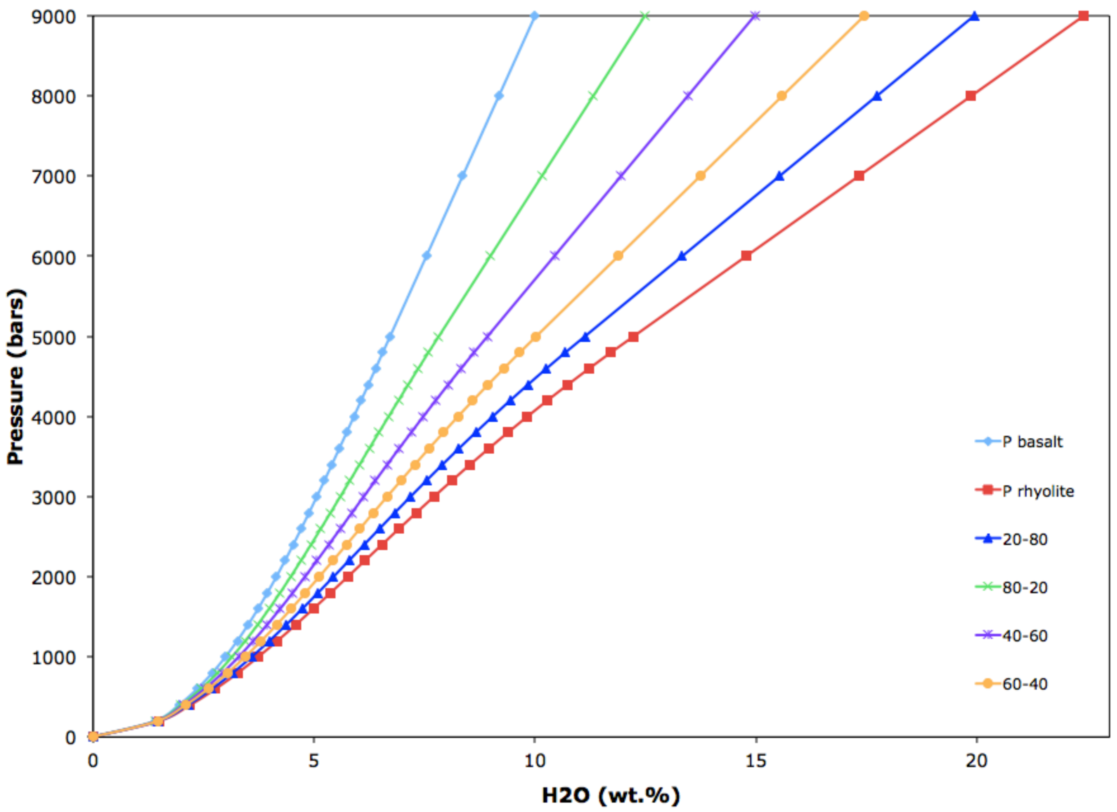

Supplementary Figure 2: *H_2O solubility relationship to pressure and melt composition calculated from VolatileCalc[29]. Solubilities for intermediate compositions are interpolated (e.g., 20-80 means 20% of basalt and 80% of rhyolite, etc.). Solubilities above 0.5 GPa up to 0.9 GPa are extrapolated. This Figure is from the Supplementary Information of ref. 28.*

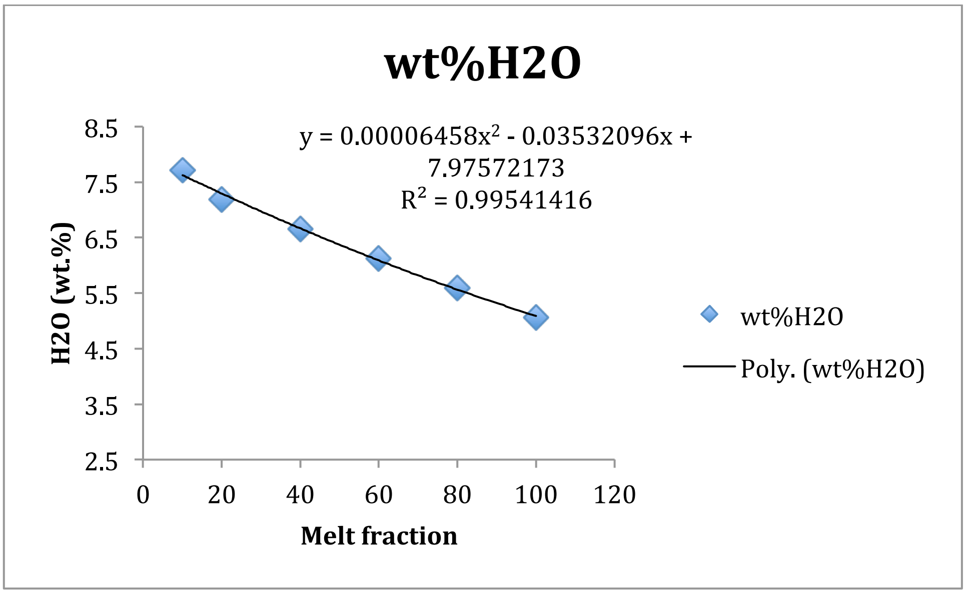

Supplementary Figure 3: *H_2O solubility is linked to melt fraction (M) by a best-fit polynomial equation. This Figure is from the Supplementary Information of ref. 28.*

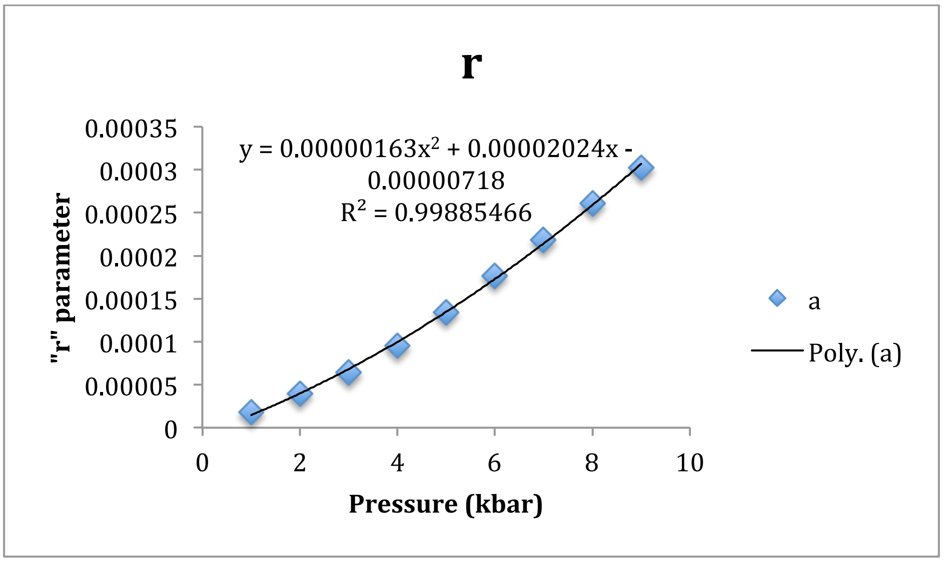

Supplementary Figure 4: *Pressure dependence of the parameter r (similar dependencies exist also for the parameters s and t) in the 2nd order polynomial equation relating the H_2O concentration to melt fraction (see equations 7, 8, 9 in the Methods section of the main text). This Figure is from the Supplementary Information of ref. 28.*

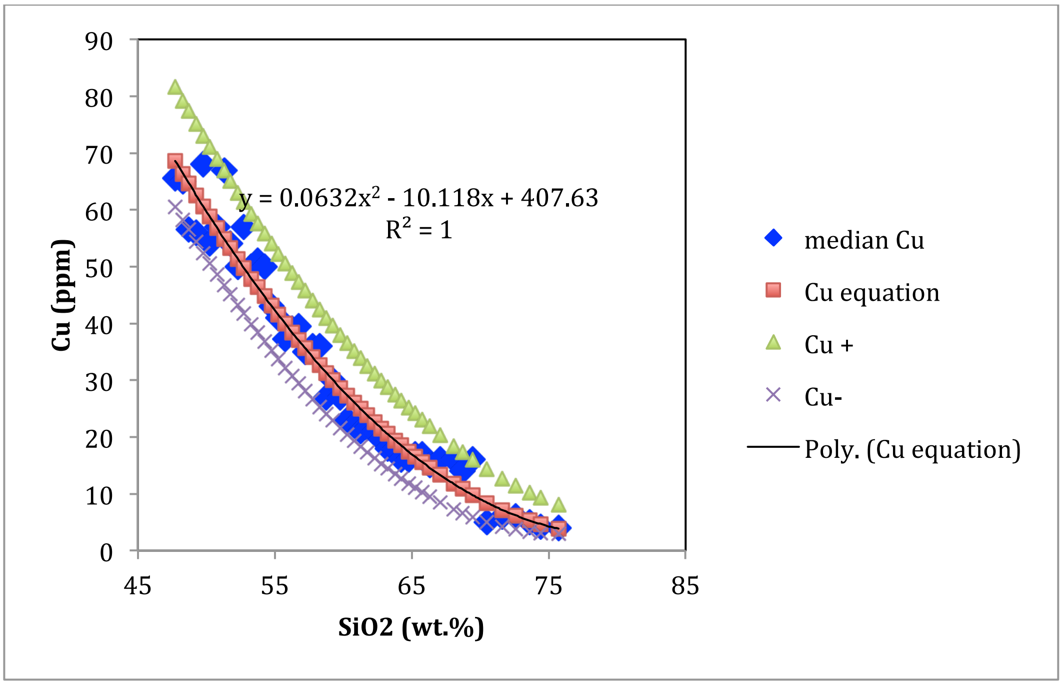

Supplementary Figure 5: *Median values of copper contents of whole magmatic rocks of thick arcs steadily decrease with SiO_2 (from Chiaradia, 2014)[30]. The upper (Cu+) and lower (Cu-) curves encompass the range of variability of the median values. Values of Cu contents within these curves are randomly changed to obtain the Cu contents in fluids exsolved from magmas with specific SiO_2 contents using random fluid-melt partition coefficients for Cu between 2 and 100. This Figure is from the Supplementary Information of ref. 28.*

Supplementary Table 1: *Relationship between lithology, SiO_2 content and melt fraction.*

Lithology	*SiO_2 (mid-value of TAS diagram[31])*	*Melt Fraction (M) (mid-value from Annen et al., 2006)[27]*
Basalt	76	0.1
Basaltic andesite	70	0.2
Andesite	63	0.34
Dacite	57	0.5
Rhyolite	49	1

Supplementary Note2

Information for the use of the Monte Carlo simulations algorithm (Supplementary Data 2)

Monte Carlo simulations have been run in 4 separate Microsoft Excel spreadsheets (see below and Supplementary Data 2) for simulation of gold and copper endowments in alkaline and calc-alkaline magmas. Two additional spreadsheets ("Alkaline GOLD&COPPER" and "Calc-alkaline GOLD&COPPER") are the combination of spreadsheets "Alkaline magmas GOLD" + "Alkaline magmas COPPER" and "Calc-alkaline magmas GOLD" + "Calc-alkaline magmas COPPER", respectively. They allow the comparison of gold and copper endowments internally to each separate system (alkaline and calc-alkaline) and can be used to reproduce, by changing the input parameters as discussed in the main text and shown in Table 1, the simulations of Figs. 2, 3, 4 in the main text. Four demo spreadsheets have been also added to show practical examples of how simulations in Figs. 2, 3, 4 were obtained.

The spreadsheet names and their functions are summarized in Supplementary Table 2.

Supplementary Table 2: *Description of the functions and outputs of the Excel spreadsheets used for Monte Carlo simulations (Supplementary Data 2).*

Spreadsheet name	*Functions*	*Outputs*
Alkaline magmas GOLD	For Monte Carlo simulations of gold endowments associated with alkaline magmas	Figs. 2b, 2d, 3a, 4a-b
Alkaline magmas COPPER	For Monte Carlo simulations of copper endowments associated with alkaline magmas	Fig. 3a, 4a
Calc-alkaline magmas GOLD	For Monte Carlo simulations of gold endowments associated with calc-alkaline magmas	Figs 2a, 2c, 3b-c, 4a-b
Calc-alkaline magmas COPPER	For Monte Carlo simulations of copper endowments associated with calc-alkaline magmas	Figs. 3b-c, 4a-b
Alkaline GOLD&COPPER	For combined simulations of gold and copper endowments in alkaline magmatic systems	Figs. 3a, 4a
Calc-alkaline GOLD&COPPER	For combined simulations of gold and copper endowments in calc-alkaline magmatic systems	Figs. 3b-c, 4a-b
Demo alkaline GOLD	Demo with reduced number of simulations for gold endowments in alkaline magmatic systems	Fig. 2b (2d)
Demo calc-alkaline GOLD	Demo with reduced number of simulations for gold endowments in calc-alkaline magmatic systems	Fig. 2a (2c)
Demo alkaline Au&Cu	Demo with reduced number of simulations for gold and copper endowments in alkaline magmatic systems	Fig. 3a
Demo calc-alkaline Au&Cu	Demo with reduced number of simulations for gold and copper endowments in calc-alkaline magmatic systems	Fig. 3b (simulations of Fig. 3c can be obtained by changing the precipitation efficiency in the related spreadsheet ("Calc-alkaline GOLD&COPPER") as discussed in the text and Methods

An Excel version 14.7.3 for Mac was used for the simulations, but the algorithm uses only hand-typed formulae and can be run in principle on any Excel version. In the attached Excel file 29

simulations (rows) are reported. By dragging down the last row of each Excel spreadsheet the number of simulations can be increased to any desired value. In this study several thousands of simulations were carried out.

Each spreadsheet consists of the color-coded groups of columns reported in Supplementary Table 3.

Supplementary Table 3: *Summary description of the color-coded groups of columns in the Excel spreadsheets of Supplementary Data 2.*

**Spreadsheets for Copper**		**Spreadsheets for Gold**	
Colour of columns	*Operations performed in columns*	*Colour of columns*	*Operations performed in columns*
Light green	Input parameters (the used values are reported in Table 1 and also below)	Light green	Input parameters (the used values are reported in Table 1 and also below)
Light orange	Calculations of crustal melt amounts according to the equations reported in Methods and Supplementary Note 2 (OUTPUT: should not be modified)	Light orange	Calculations of crustal melt amounts according to the equations reported in Methods and Supplementary Note 2 (OUTPUT: should not be modified)
Light blue	Calculations of residual melt amounts, SiO_2 and Cu contents according to the equations reported in Methods and Supplementary Note 2 (OUTPUT: should not be modified)	Light blue	Calculations of residual melt amounts (OUTPUT: should not be modified)
Light red	Calculations of the total melt amounts according to the equations reported in Methods and Supplementary Note 2 (OUTPUT: should not be modified)	Light red	Calculations of the total melt amounts according to the equations reported in Methods and Supplementary Note 2 (OUTPUT: should not be modified)
Blue	Calculations of the H_2O contents in the residual melt according to the equations reported in Methods and Supplementary Note 2 (should not be modified)	Blue	Calculations of the H_2O contents in the residual melt according to the equations reported in Methods and Supplementary Note 2 (should not be modified)
Orange	Calculations of the H_2O contents in the residual melt according to the equations reported in Methods and Supplementary Note 2 (OUTPUT: should not be modified)	Orange	Calculations of the H_2O contents in the residual melt according to the equations reported in Methods and Supplementary Note 2 (OUTPUT: should not be modified)
Red	Calculations of the H_2O contents in the total melt according to the equations reported in Methods and Supplementary Note 2 (OUTPUT: should not be modified)	Red	Calculations of the H_2O contents in the total melt according to the equations reported in Methods and Supplementary Note 2 (OUTPUT: should not be modified)
Green	Cu endowment calculations (OUTPUT: should not be modified)	Yellow	Au endowment calculations (OUTPUT: should not be modified)

Each group of columns is described below, whereas a detailed description of all parameters of the algorithm is provided in Supplementary Tables 5-6 below.

Description of the groups of columns

Input parameters

These parameters have the input values (fixed or randomly variable) reported in Supplementary Table 4 below, which is the same as Table 1 in the main text.

Supplementary Table 4: *Values used for input parameters in the model for an injection rate of 5 mm year^{-1} of a basaltic melt at 1200°C through a disk of 7500 m radius[a] (equivalent to a magma flux of 0.0009 km^3 year^{-1}), into a crust characterized by a geothermal gradient of 20°C km^{-1} (ref. 27).*

Input parameter	Value(s)
Time	Random between 0 and 5 Ma
Pressure (calc-alkaline systems)	Random between 0.15 and 0.9 GPa
Pressure (alkaline systems)	Random between 0.15 and 0.6 GPa
H_2O in parent magma	Random between 2 and 4 wt.% [32]
H_2O in crustal rocks	Random between 0.2 and 1 wt.% [28]
Fluid-melt partition coefficient of copper	Random between 2 and 100 [28]
Fluid-melt partition coefficient of gold	Random between 10 and 100 [33]
Gold content in calc-alkaline magmas	Random between 6 and 9 ppb [34]
Gold content in alkaline magmas	Random between 10 and 32 ppb [35]
Copper content in calc-alkaline/alkaline magmas	Constrained through SiO_2-Cu relationship of alkaline and calc-alkaline magmas[30,36]
Cu precipitation efficiency in Cu-rich calc-alkaline systems	50% (Figs. 3a-c, Fig. 4a-b)
Cu precipitation efficiency in Au-rich calc-alkaline systems	30% (Fig. 4b)
Cu precipitation efficiency in Au-rich alkaline systems	50% (Fig. 3a, 4a)
Au precipitation efficiency in Cu-rich calc-alkaline systems	50% (Fig. 2a, 3b-c); 0.67% (Figs. 4a-b)
Au precipitation efficiency in Au-rich calc-alkaline systems	5% (Fig. 4b)
Au precipitation efficiency in Au-rich alkaline systems	50% (Figs. 2b, 3a); 3.3% (Fig. 4a)

[a] an average size for crustal magma chambers, typically ranging between 5000 and 10000 m (ref. 37).

Calculations of crustal melt amounts (output)

These columns report calculations of the crustal melt amounts as parameterized from results of Annen et al. (2006)[27] (Supplementary Figure 1). Logical tests columns eliminate the values that returned impossible solutions based on the mathematical parameterization (see details of each column in Supplementary Tables 5-6 below). Crustal melts are those produced by partial melting of the crustal rocks due to continuous injection of basaltic melt at 1200°C at variable crustal depths and at a fixed average rate of 5 mm year^{-1} after a certain incubation time defined as the time from onset of basaltic melt injection until the first appearance of partial melting of the crustal rocks.

Calculations of residual melt amounts (output)

These columns report calculations of the melt melt amounts as parameterized from results of Annen et al. (2006)[27] (Supplementary Figure 1). Logical tests columns eliminate the values that returned impossible solutions based on the mathematical parameterization (see details of each column in Supplementary Tables 5-6 below). Residual melts are those formed from fractional crystallization of the basaltic melt injected at 1200°C at variable crustal depths and at a fixed

average rate of 5 mm year^{-1} after a certain incubation time defined as the time from onset of basaltic melt injection until the first appearance of residual melt.

Calculations of total melt amounts (output)

These columns report calculations relative to amount (Supplementary Figure 1) and composition (SiO_2: see Supplementary Table 1) of the hybrid melt resulting from the sum of the two melts above.

Calculations of the H_2O contents in the residual melt (output)

These columns report calculations of the concentrations, mass and volume of H_2O in residual melts. These calculations are based on the pressure and melt composition dependence of H_2O solubility parameterized from the H_2O solubility model of Newman and Lowenstern (2001)[29] (Supplementary Figures 2-5). According to pressure and melt composition, H_2O can be either in solution or in excess in the silicate melts of the simulations. Logical tests are performed to eliminate impossible solutions as detailed in Supplementary Tables 5-6 below.

Calculations of the H_2O contents in the crustal melt (output)

These columns report calculations of the concentrations, mass and volume of H_2O in crustal melts. These calculations are based on the pressure and melt composition dependence of H_2O solubility parameterized from the H_2O solubility model of Newman and Lowenstern (2001) [29] (Supplementary Figures 2-5). According to pressure and melt composition, H_2O can be either in solution or in excess in the silicate melts of the simulations. Logical tests are performed to eliminate impossible solutions as detailed in Supplementary Tables 5-6 below.

Calculations of the H_2O contents in the total melt (output)

These columns report calculations of the concentrations, mass and volume of H_2O in the total hybrid melts.

Copper and gold endowment calculations (output)

These columns report calculations of the metal endowment associated with each simulation based on the amount of exsolvable H_2O, metal contents in melts and fluid-melt partition coefficient values (Supplementary Table 4).

Data Plot

Selected Monte Carlo outputs (Figs. 1-4 in the main text) were extracted from the Monte Carlo simulation Excel spreadsheets into a separate Excel spreadsheet (see demo spreadsheets in the Supplementary Data 2). Excel cells that returned impossible solutions (i.e., "False" in logical tests, "#DIV/0!" in calculation cells) were discarded. The ioGAS software was used to plot the selected outputs and obtain the statistical information reported in Figs. 1-4 in the main text.

***Supplementary Table 5**: Description of the parameters used in Monte Carlo simulations for melt injection rate of 5 mm year^{-1} through a disk of 7500 m radius (equivalent to 0.0009 km^3 year^{-1}) using the spreadsheets "alkaline magmas GOLD" and "calc-alkaline magmas GOLD" in the Supplementary Data 2.*

Column in excel spreadsheet	*Label of column in excel spreadsheet*	*Explanation*
A	Time	time since first injection starts (random between 0 and 5 Ma)
B	H_2O parent	wt.% content of H_2O in parent magma (random between 2 and 4 wt.%)
C	H_2O assimilant	wt.% content of H_2O in assimilant crust (random between 0.2 and 1 wt.%)
D	P (kbars)	pressure at which magma accumulation occurs (random between 1.5 and 9 kbar)
E	Magma rate	fixed magma rate at 5 mm/year (the magma rate used by Annen et al. (2006)[27] for the parameterization of the crustal and residual melt fractions produced at different depths).
F	Magma rate time integrated	magma rate integrated over the random time of the same row.
G	Disk radius	fixed radius of 7500 m for a circular shape section through which the above flux rate is integrated. This radius is typical of crustal batholiths (Annen, 2009)[37]. The integrated magma volume corresponds to 0.0009 km^3/year, which is a typical average long-term flux for arc magmatism.
H	Magma rate (tons/Ma/km arc)	fixed magma rate at 5 mm/year (the magma rate used by Annen et al., 2006[27], for the parameterization of the crustal and residual melt fractions produced at different depths).
I	Magma rate time integrated (tons/Ma/km arc)	magma rate integrated over the random time of the same row
J	Kd	Au KD between melt and fluid random values between 10 and 100[33]
K	Au total melt ppm	Random range of Au contents in melt: 0.006-0.009 ppm in calc-alkaline magmas and 0.010-0.032 ppm in alkaline magmas (see Table A)
L	x crustal	parameter "x" of the second order equation ($y=ax^2+bx+c$) describing the dependence on time of crustal melt fraction produced at a random depth from parameterization of Annen et al. (2006) [27].
M	y crustal	parameter "y" of the second order equation ($y=ax^2+bx+c$) describing the dependence on time of crustal melt fraction produced at a certain depth from parameterization of Annen et al. (2006) [27].
N	z crustal	parameter "z" of the second order equation ($y=ax^2+bx+c$) describing the dependence on time of crustal melt fraction produced at a certain depth from parameterization of Annen et al. (2006) [27].
O	Crustal melt fraction Annen	crustal melt fraction parameterized from Annen et al. (2006) [27] model.
P	Crustal melt fraction Annen logical test	logical test for crustal melt fraction calculated from Annen et al. (2006) [27] model: if the parameterized value is <0 → 0, otherwise is the parameterized value.
Q	Crustal melt fraction Annen logical test 2	logical test 2 for crustal melt fraction calculated from Annen et al. (2006) [27] model to avoid negative values below 1 Ma.
R	Crustal melt fraction Annen logical test 3	logical test 3 for crustal melt fraction calculated from Annen et al. (2006) [27] model to avoid negative values below 0.25 Ma.
S	Volume of crustal melt integrated over time	volume of crustal melt produced by basaltic melt passing through a circle with radius equal to 7500 m at the depth value (pressure) of the same simulation row.
T	Tons of crustal melt time integrated	the above volume multiplied by an average andesitic melt density of 2.6 gm/cm^3. (Although density changes with melt composition we have assumed for simplicity an average andesitic melt density for melts ranging from basaltic to rhyolitic).
U	Crustal melt% Annen	% of crustal melt fraction from parameterization of Annen et al. (2006) [27].

V	x residual	parameter “x” of the second order equation ($y=ax^2+bx+c$) describing the dependence on time of residual melt fraction produced at a random depth from parameterization of Annen et al. (2006) [27].
W	y residual	parameter “y” of the second order equation ($y=ax^2+bx+c$) describing the dependence on time of residual melt fraction produced at a random depth from parameterization of Annen et al. (2006) [27].
X	z residual	parameter “z” of the second order equation ($y=ax^2+bx+c$) describing the dependence on time of residual melt fraction produced at a random depth from parameterization of Annen et al. (2006) [27].
Y	residual Melt fraction Annen	residual melt fraction parameterized from Annen et al. (2006)[27] model
Z	residual Melt fraction Annen logical test	logical test for residual melt fraction parameterized from Annen et al. (2006) [27] model to avoid negative values below 0.75 Ma.
AA	residual Melt fraction Annen logical test2	logical test 2 for residual melt fraction parameterized from Annen et al. (2006) [27] model to avoid negative values below 0.5 Ma.
AB	V residual melt fraction time integrated	volume of residual melt produced by basaltic melt passing through a circle with radius equal to 7500 m at the depth value (pressure) of the same simulation row.
AC	tons residual melt fraction time integrated	the above volume multiplied by an average andesitic melt density of 2.6 gm/cm^3. (Although density changes with melt composition we have assumed for simplicity an average andesitic melt density for melts ranging from basaltic to rhyolitic).
AD	residual Melt% Annen	% of residual melt parameterized from Annen et al. (2006) [27]
AE	all melt %	sum of residual and crustal melts percentages
AF	Total melt (tons)	tons of total melt (residual + crustal)
AG	Volume km^3 total melt	volume (km^3) of total melt
AH	M res hydrous melt ton	tons of residual hydrous melt (this is equivalent to the tons of undersaturated melt)
AI	Volume km^3 hydrous melt	volume (km^3) of hydrous melt
AJ	H_2O wt% in residual melt	H_2O wt.% concentration in the residual melt from random initial concentration (H_2O_{in}=2-4 wt.%) assuming a completely incompatible behavior for H_2O during magma fractionation ($=100*H_2O_{in}*F^{-1}$, where F is the residual melt fraction)
AK	log test H_2O wt% in residual melt	H_2O wt.% in residual melt assuming that the minimum residual melt remaining after fractionation is 10%
AL	r H_2O	parameter “r” of the second order equation ($y=ax^2+bx+c$) describing the dependence on pressure of the H_2O content of a silicate melt of a random composition (=melt fraction) paramaterized from the P-X dependency of H_2O solubility of VolataileCalc (Newman and Lowenstern, 2002)[29]
AM	s H_2O	parameter “s” of the second order equation ($y=ax^2+bx+c$) describing the dependence on pressure of the H_2O content of a silicate melt of a random composition (=melt fraction) paramaterized from the P-X dependency of H_2O solubility of VolataileCalc (Newman and Lowenstern, 2002) [29]
AN	t H_2O	parameter “t” of the second order equation ($y=ax^2+bx+c$) describing the dependence on pressure of the H_2O content of a silicate melt of a random composition (=melt fraction) paramaterized from the P-X dependency of H_2O solubility of VolataileCalc (Newman and Lowenstern, 2002) [29]
AO	residual melt Annen logical test for H_2O solubility (F=100-10%)	logical test for residual melt fraction parameterized from Annen et al. (2006) [27] model to avoid residual melt fractions <10%
AP	H_2O solubility in melt (F =100-10 %)	H_2O solubility in the residual melt at the random pressure and residual melt fraction (logical test corrected) of the same raw
AQ	H_2O wt% in residual melt logical test 100-	effective H_2O wt% content of the residual melt corrected for oversaturation (i.e., if the theoretical amount of H_2O in the residual melt is higher than the maximum permissible H_2O content in that

	10% F range	melt composition and at that specific P, then the latter value is taken as the effective H_2O content of that simulation and the remainder H_2O is in excess and must have been exsolved).
AR	Excess residual H_2O	H_2O in excess according to the above explanation
AS	H_2O residual (tons)	tons of H_2O dissolved in residual melt
AT	Log test excess residual H_2O tons	tons of excess H_2O (logical test to avoid negative values, if negative means that excess H_2O is 0)
AU	Excess H_2O residual (tons)	tons of excess H_2O from residual melt (H_2O that has been liberated by the residual melt at specific P, time and composition values occurring in the same raw)
AV	H_2O wt% in partial melt	H_2O wt.% concentration in the crustal melt from random initial concentration (H_2O_{in}=0.2-1 wt.%) assuming a completely incompatible behavior for H_2O during partial melting (=100*H_2O_{in}*F^{-1}, where F is the crustal melt fraction)
AW	H_2O solubility (wt.%) with Annen crustal melt %	H_2O solubility in the crustal melt at the random pressure and crustal melt fraction (logical test corrected) of the same raw
AX	H_2O solubility (wt.%) with Annen crustal melt % logical test	effective H_2O wt% content of the crustal melt corrected for oversaturation (i.e., if the theoretical amount of H_2O in the crustal melt is higher than the maximum permissible H_2O content in that melt composition and at that specific P, then the latter value is taken as the effective H_2O content of that simulation and the remainder H_2O is in excess and must have been exsolved).
AY	Excess H_2O partial melt	H_2O in excess according to the above explanation
AZ	H_2O crustal (tons)	tons of H_2O dissolved in crustal melt
BA	Excess H_2O crustal (tons)	tons of H_2O liberated by the crustal melt at specific P, time and composition values occurring in the same raw
BB	log test excess crustal H_2O tons	tons of excess H_2O (logical test to avoid negative values, if negative means that excess H_2O is 0)
BC	H_2O total in melt (tons)	sum of tons of H_2O dissolved in the residual and crustal melt
BD	H_2O% in hydrous melt	wt% of H_2O in over or undersaturated hydrous melt
BE	exsolvable H_2O tons	tons of H_2O dissolved in residual hydrous melt
BF	Excess H_2O total in melt (tons)	sum of the tons of excess H_2O liberated by both the crustal and residual melt
BG	Excess total H_2O NEW log test	sum of the tons of excess H_2O liberated by both the crustal and residual melt avoiding negative values (logical tests for excess H_2O in crustal and residual melts)
BH	M melt - excess H_2O tons	mass in tons of the total melt (residual + crustal) minus the sum of excess H_2O (residual + crustal)
BI	% H_2O excess	wt% of total excess H_2O
BJ	Moles of excess H_2O	moles of H_2O exsolved from oversaturated melts
BK	Au tot ton	tons of Au in melt
BL	Au ppm res melt	ppm of Au in H_2O-saturated melt (where part of the Au has been lost to the exsolved H_2O)
BM	Au ppm fluid	ppm Au in the exsolved fluid of the oversaturated melt
BN	Au tot res melt Mt	Mt of Au in the "residual" oversaturated melt (this is identical to the total Au amount of melt only in undersaturated melts)
BO	Au tot in excess fluid Mt	Mt of Au that are lost by the oversaturated melt to the exsolved fluid
BP	50% Au tot in excess fluid Mt	50% of Mt of Au that are lost by the oversaturated melt to the exsolved fluid
BQ	Au tot Mt	Mt of Au in melt
BR	Au tot ton	tons of Au in hydrous under and oversaturated melt
BS	Au res melt	ppm of Au in the hydrous melt after it exsolved H_2O at the saturation P
BT	Au fluid	ppm of Au in the fluid exsolved at saturation depth
BU	Au tot res melt Mt	Mt of Au in the hydrous melt after H_2O exsolution
BV	Au tot in exsolvable	tons of Au in exsolvable fluid at saturation depth

	fluid tons	
BW	Au tot t	tons of Au total in hydrous melt (melt + exsolved fluid)
BX	Precipitation efficiency for Au	Precipitation efficiency of Au in % (can be changed to any value)
BY	PE Au fluid tons	tons of Au in exsolvable fluid at saturation depth recalculated according to precipitation efficiency

Supplementary Table 6*: Description of the parameters used in Monte Carlo simulations for melt injection rate of 5 mm year^{-1} through a disk of 7500 m radius (equivalent to 0.0009 km^3 year^{-1}) using the spreadsheets "alkaline magmas COPPER" and "calc-alkaline magmas COPPER" in the Supplementary Data 2.*

Column in excel spreadsheet	*Label of column in excel spreadsheet*	*Explanation*
A	Time	time since first injection starts (random between 0 and 5 Ma)
B	H_2O parent	wt.% content of H_2O in parent magma (random between 2 and 4 wt.%)
C	H_2O assimilant	wt.% content of H_2O in assimilant crust (random between 0.2 and 1 wt.%)
D	P (kbars)	pressure at which magma accumulation occurs (random between 1.5 and 9 kbar)
E	Magma rate	fixed magma rate at 5 mm/year (the magma rate used by Annen et al. (2006) [27] for the parameterization of the crustal and residual melt fractions produced at different depths).
F	Magma rate time integrated	magma rate integrated over the random time of the same row.
G	Disk radius	fixed radius of 7500 m for a circular shape section through which the above flux rate is integrated. This radius is typical of crustal batholiths (Annen, 2009)[37]. The integrated magma volume corresponds to 0.0009 km^3/year, which is a typical average long-term flux for arc magmatism.
H	Magma rate (tons/Ma/km arc)	fixed magma rate at 5 mm/year (the magma rate used by Annen et al., 2006[27], for the parameterization of the crustal and residual melt fractions produced at different depths).
I	Magma rate time integrated (tons/Ma/km arc)	magma rate integrated over the random time of the same row
J	Kd	Cu KD between melt and fluid random values between 2 and 100[28]
K	x crustal	parameter "x" of the second order equation ($y=ax^2+bx+c$) describing the dependence on time of crustal melt fraction produced at a random depth from parameterization of Annen et al. (2006) [27].
L	y crustal	parameter "y" of the second order equation ($y=ax^2+bx+c$) describing the dependence on time of crustal melt fraction produced at a certain depth from parameterization of Annen et al. (2006) [27].
M	z crustal	parameter "z" of the second order equation ($y=ax^2+bx+c$) describing the dependence on time of crustal melt fraction produced at a certain depth from parameterization of Annen et al. (2006) [27].
N	Crustal melt fraction Annen	crustal melt fraction parameterized from Annen et al. (2006)[27] model.
O	Crustal melt fraction Annen logical test	logical test for crustal melt fraction calculated from Annen et al. (2006) [27] model: if the parameterized value is <0 → 0, otherwise is the parameterized value.
P	Crustal melt fraction Annen logical test 2	logical test 2 for crustal melt fraction calculated from Annen et al. (2006) [27] model to avoid negative values below 1 Ma.
Q	Crustal melt fraction Annen logical test 3	logical test 3 for crustal melt fraction calculated from Annen et al. (2006) [27] model to avoid negative values below 0.25 Ma.
R	Volume of crustal melt integrated over time	volume of crustal melt produced by basaltic melt passing through a circle with radius equal to 7500 m at the depth value (pressure) for the same simulation row.
S	Tons of crustal melt time integrated	the above volume multiplied by an average andesitic melt density of 2.6 gm/cm^3. (Although density changes with melt composition we have assumed for simplicity an average andesitic melt density for melts ranging from basaltic to rhyolitic).
T	Crustal melt% Annen	% of crustal melt fraction from parameterization of Annen et al. (2006) [27].
U	x residual	parameter "x" of the second order equation ($y=ax^2+bx+c$) describing the dependence on time of residual melt fraction produced at a random depth from parameterization of Annen et al. (2006) [27].

V	y residual	parameter "y" of the second order equation ($y=ax^2+bx+c$) describing the dependence on time of residual melt fraction produced at a random depth from parameterization of Annen et al. (2006) [27].
W	z residual	parameter "z" of the second order equation ($y=ax^2+bx+c$) describing the dependence on time of residual melt fraction produced at a random depth from parameterization of Annen et al. (2006) [27].
X	residual Melt fraction Annen	residual melt fraction parameterized from Annen et al. (2006)[27] model
Y	residual Melt fraction Annen logical test	logical test for residual melt fraction parameterized from Annen et al. (2006) [27] model to avoid negative values below 0.75 Ma.
Z	residual Melt fraction Annen logical test2	logical test 2 for residual melt fraction parameterized from Annen et al. (2006) [27] model to avoid negative values below 0.5 Ma.
AA	V residual melt fraction time integrated	volume of residual melt produced by basaltic melt passing through a circle with radius equal to 7500 m at the depth value (pressure) for the same row.
AB	tons residual melt fraction time integrated	the above volume multiplied by an average andesitic melt density of 2.6 gm/cm^3. (Although density changes with melt composition we have assumed for simplicity an average andesitic melt density for melts ranging from basaltic to rhyolitic).
AC	residual Melt% Annen	% of residual melt parameterized from Annen et al. (2006) [27]
AD	all melt %	sum of residual and crustal melts percentages
AE	M total melt ton	total tons of residual + crustal melts
AF	Volume km^3 total melt	volume (km^3) of total melt
AG	M res hydrous melt ton	tons of residual hydrous melt (this is equivalent to the tons of undersaturated melt)
AH	Volume km^3 hydrous melt	volume (km^3) of hydrous melt
AI	SiO_2 total	SiO_2 relationship to melt fraction (F) based on the empirical equation [SiO_2 $35.436F^2 - 68.859F + 82.439$]
AJ	a Cu	a parameter of the second order equation ($y=ax^2+bx+c$, where y Cu (ppm) and x SiO_2 (wt.%)) describing the Cu concentration variability at any SiO_2 value parameterized from the median values of Cu and SiO_2 from continental arc rocks (from Chiaradia, 2014)[30].
AK	b Cu	b parameter of the second order equation ($y=ax^2+bx+c$, where y Cu (ppm) and x SiO_2 (wt.%)) describing the Cu concentration variability at any SiO_2 value parameterized from the median values of Cu and SiO_2 from continental arc rocks (from Chiaradia, 2014) [30].
AL	c Cu	c parameter of the second order equation ($y=ax^2+bx+c$, where y Cu (ppm) and x SiO_2 (wt.%)) describing the Cu concentration variability at any SiO_2 value parameterized from the median values of Cu and SiO_2 from continental arc rocks (from Chiaradia, 2014) [30].
AM	Cu ppm	Cu content of melt (ppm)
AN	H_2O wt% in residual melt	H_2O wt.% concentration in the residual melt from random initial concentration (H_2O_{in}=2-4 wt.%) assuming a completely incompatible behavior for H_2O during magma fractionation ($=100*H_2O_{in}*F^{-1}$, where F is the residual melt fraction)
AO	residual melt Annen logical test for H_2O solubility (F=100-10%)	logical test for residual melt fraction parameterized from Annen et al. (2006) [27] model to avoid residual melt fractions <10%
AP	log test H_2O wt% in residual melt	H_2O wt.% in residual melt assuming that the minimum residual melt remaining after fractionation is 10%
AQ	r H_2O	parameter "r" of the second order equation ($y=ax^2+bx+c$) describing the dependence on pressure of the H_2O content of a silicate melt of a random composition (=melt fraction) paramaterized from the P-X dependency of H_2O solubility of VolataileCalc (Newman and Lowenstern, 2002) [29]
AR	s H_2O	parameter "s" of the second order equation ($y=ax^2+bx+c$) describing the dependence on pressure of the H_2O content of a silicate melt of a random composition (=melt fraction) paramaterized from the P-X dependency of H_2O solubility of VolataileCalc (Newman and

		Lowenstern, 2002) [29]
AS	t H_2O	parameter "t" of the second order equation ($y=ax^2+bx+c$) describing the dependence on pressure of the H_2O content of a silicate melt of a random composition (=melt fraction) paramaterized from the P-X dependency of H_2O solubility of VolataileCalc (Newman and Lowenstern, 2002) [29]
AT	H_2O solubility in melt (F =100-10 %)	H_2O solubility in the residual melt at the random pressure and residual melt fraction (logical test corrected) of the same raw
AU	H_2O wt% in residual melt logical test 100-10% F range	effective H_2O wt% content of the residual melt corrected for oversaturation (i.e., if the theoretical amount of H_2O in the residual melt is higher than the maximum permissible H_2O content in that melt composition and at that specific P, then the latter value is taken as the effective H_2O content of that simulation and the remainder H_2O is in excess and must have been exsolved).
AV	H_2O residual (tons)	tons of H_2O dissolved in residual melt
AW	Excess residual H_2O	H_2O in excess according to the above explanation
AX	Excess H_2O residual (tons)	tons of excess H_2O from residual melt (H_2O that has been liberated by the residual melt at specific P, time and composition values occurring in the same raw)
AY	Log test excess residual H_2O tons	tons of excess H_2O (logical test to avoid negative values, if negative means that excess H_2O is 0)
AZ	H_2O wt% in partial melt	H_2O wt.% concentration in the crustal melt from random initial concentration (H_2O_{in}=0.2-1 wt.%) assuming a completely incompatible behavior for H_2O during partial melting ($=100*H_2O_{in}*F^{-1}$, where F is the crustal melt fraction)
BA	H_2O solubility (wt.%) with Annen crustal melt %	H_2O solubility in the crustal melt at the random pressure and crustal melt fraction (logical test corrected) of the same raw
BB	H_2O solubility (wt.%) with Annen crustal melt % logical test	effective H_2O wt% content of the crustal melt corrected for oversaturation (i.e., if the theoretical amount of H_2O in the crustal melt is higher than the maximum permissible H_2O content in that melt composition and at that specific P, then the latter value is taken as the effective H_2O content of that simulation and the remainder H_2O is in excess and must have been exsolved).
BC	Excess H_2O partial melt	H_2O in excess according to the above explanation
BD	H_2O crustal (tons)	tons of H_2O dissolved in crustal melt
BE	Excess H_2O crustal (tons)	tons of H_2O liberated by the crustal melt at specific P, time and composition values occurring in the same raw
BF	log test excess crustal H_2O tons	tons of excess H_2O (logical test to avoid negative values, if negative means that excess H_2O is 0)
BG	H_2O total in melt (tons)	sum of tons of H_2O dissolved in the residual and crustal melt
BH	Excess H_2O total in melt (tons)	sum of the tons of excess H_2O liberated by both the crustal and residual melt
BI	Excess total H_2O NEW log test	sum of the tons of excess H_2O liberated by both the crustal and residual melt avoiding negative values (logical tests for excess H_2O in crustal and residual melts)
BJ	% H_2O excess	wt% of total excess H_2O
BK	Moles of excess H_2O	moles of H_2O exsolved from oversaturated melts
BL	H_2O% in hydrous melt	wt% of H_2O in over or undersaturated hydrous melt
BM	H_2O % in melt	wt.% content of H_2O in the melt
BN	exsolvable H_2O tons	tons of H_2O dissolved in residual hydrous melt
BO	Cu tot ton	tons of Cu in melt
BP	Cu ppm res melt	ppm of Cu in H_2O-saturated melt (where part of the Cu has been lost to the exsolved H_2O)
BQ	Cu ppm fluid	ppm Cu in the exsolved fluid of the oversaturated melt
BR	Cu tot res melt Mt	Mt of Cu in the "residual" oversaturated melt (this is identical to the total Cu amount of melt only in undersaturated melts)

BS	Cu tot in excess fluid Mt	Mt of Cu that are lost by the oversaturated melt to the exsolved fluid
BT	50% Cu tot in excess fluid Mt	50% of Mt of Cu that are lost by the oversaturated melt to the exsolved fluid
BU	Cu tot Mt in melt	Mt of Cu in melt
BV	Cu tot ton in hydrous melt	tons of Cu in hydrous under and oversaturated melt
BW	Cu res melt (ppm)	ppm of Cu in the hydrous melt after it exsolved H_2O at the saturation P
BX	Cu fluid (ppm)	ppm of Cu in the fluid exsolved at saturation depth
BY	Cu tot res melt Mt	Mt of Cu in the hydrous melt after H_2O exsolution
BZ	Cu tot in exsolvable fluid Mt	Mt of Cu in exsolvable fluid at saturation depth
CA	Cu tot Mt	Mt of Cu total in hydrous melt (melt + exsolved fluid)
CB	Precipitation efficiency	Precipitation efficiency of Cu: default is 50%
CC	50% Cu fluid Mt	Mt of Cu in exsolvable fluid recalculated for precipitation efficiency

Supplementary References

[1] Buret, Y. et al. From a long-lived upper-crustal magma chamber to rapid porphyry copper emplacement: Reading the geochemistry of zircon crystals at Bajo de la Alumbrera (NW Argentina). *Earth Planet. Sci. Lett.* **450**, 120-131 (2016).
[2] Garwin, S., The geologic setting of intrusion-related hydrothermal systems near the Batu Hijau porphyry copper-gold deposit, Sumbawa, Indonesia. *Society of Economic Geologists Special Publication* **9**, 333–366 (2002).
[3] Braxton, D. P. & Cooke, D. R., From crucible to graben in 2.3 Ma: A high-resolution geochronological study of porphyry life cycles, Boyongan-Bayugo copper-gold deposits, Philippines. *Geology* **40**, 471–47 (2012).
[4] Von Quadt, A. et al. Zircon crystallization and the lifetimes of ore-forming magmatic-hydrothermal systems. *Geology* **39**, 731–734 (2011).
[5] Dilles, J. H., Stein, H. J. & Martin, M. W. Re-Os and U-Pb ages for the duration of the giant Butte, Montana, porphyry Cu-Mo and Cordilleran base metal lode ore deposit. *IAVCEI General Assembly 2004*, Pucon, Chile (2004).
[6] Schutte, P., Chiaradia, M. & Beate, B. Petrogenetic Evolution of Arc Magmatism Associated with Late Oligocene to Late Miocene Porphyry-Related Ore Deposits in Ecuador. *Econ. Geol.* **105**, 1243-1270 (2010).
[7] Barra, F. *et al.*, Timing and formation of porphyry Cu–Mo mineralization in the Chuquicamata district, northern Chile: new constraints from the Toki cluster. *Mineralium Deposita* **48**, 629–651 (2013).
[8] Ballard, J. R., Palin, J. M., Williams, I. S. & Campbell, I. H. Downloaded from geology.gsapubs.org on February 7, 2014 Two ages of porphyry intrusion resolved for the super-giant Chuquicamata copper deposit of northern Chile by ELA-ICP-MS and SHRIMP. *Geology* **29**, 383-386 (2001).
[9] Chelle-Michou, C., Chiaradia, M., Ulianov, A. & Beguelin, P. Petrologic evolution of the magmatic suite associated with the Coroccohuayco Cu(-Au-Fe) porphyry-skarn deposit, Peru. *J. Pet* **56**, 1829–1862 (2015).
[10] Correa, K. J., Rabbia, O. M., Hernández, L. B., Selby, D. & Astengo, M. The timing of magmatism and ore formation in the El Abra porphyry copper deposit, northern Chile: Implications for long-lived multiple-event magmatic-hydrothermal porphyry systems. *Econ. Geol.* **111**, 1-28 (2016).
[11] Zimmermann, A., Stein, H.J., Morgan, J.W., Markey, R.J. & Watanabe, Y. Re–Os geochronology of the El Salvador porphyry Cu–Mo deposit, Chile: Tracking analytical improvements in accuracy and precision over the past decade. *Geochim. Cosmochim. Acta* **131**, 13-32 (2014).
[12] Maksaev, V. et al. New chronology for El Teniente, Chilean Andes, from U-Pb, $^{40}Ar/^{39}Ar$, Re-Os, and fission-track dating: implications for the evolution of a supergiant porphyry Cu-Mo deposit. In *Andean metallogeny: new discoveries, concepts and updates* (eds. Sillitoe, R. H., Perello, J. & Vidal, C. E.), Society of Economic Geologists, *SEG Special Publication* **11**, 15–54 (2004).
[13] Cannell, J., Cooke, D. R., Walshe, J. L. & Stein, H. Geology, mineralization, alteration and structural evolution of the El Teniente porphyry Cu–Mo deposit. *Econ. Geol.* **100**, 979–1003 (2005).
[14] Von Quadt, A. et al. The Elatsite porphyry copper deposit in the Panagyurishte ore district, Srednogorie zone, Bulgaria: U-Pb zircon geochronology and isotope-geochemical investigations of magmatism and ore genesis. *Geological Society, London, Special Publications* **204**, 119-135 (2002).

[15] Hedenquist, J. W., Arribas, A. & Reynolds, T. J. Evolution of an intrusion-centered hydrothermal system; Far Southeast-Lepanto porphyry and epithermal Cu-Au deposits, Philippines. *Econ. Geol.* **93**, 373-404 (1998).
[16] Rinne, M. L. et al. Geology and Geochronology of the Golpu Porphyry and Wafi Epithermal Deposit, Morobe Province, Papua New Guinea. *Econ. Geol.* **113**, 271-294 (2018).
[17] Wafforn, S. Geo- and Thermochronology of the Ertsberg-Grasberg Cu-Au Mining District, west New Guinea, Indonesia. Unpublished PhD Thesis, The University of Texas at Austin, 356 pp. (2017).
[18] Pollard, P. J., Taylor, R. G. & Peters, L. Ages of intrusion, alteration, and mineralization at the Grasberg Cu-Au deposit, Papua, Indonesia. *Economic Geology* **100**, 1005-1020 (2005).
[19] Baker, T. et al. The geology of the Kisladag porphyry gold deposit, Turkey. *Society of Economic Geologists Special Publication* **19**, 1-27 (2016).
[20] Paolillo, L. Petrochronology of zircons from magmatic rocks of the Kisladag Au(-Mo) porphyry deposit (western Anatolia, Turkey). Unpublished MSc Thesis, University of Geneva (Switzerland), 187 pp. (2018).
[21] Stein, H., Markey, R., Sillitoe, R. & Perello, J. Defining the lifespan of a Giant Porphyry Cu Deposit: Re-Os Dating at Los Pelambres, Chile. Geochim. Cosmochim. Acta **66-Supplement 1**, 738 (2002).
[22] Large, S. J. E., von Quadt, A., Wotzlaw, J.-F., Guillong, M. & Heinrich, C. A. Magma Evolution Leading to Porphyry Au-Cu Mineralization at the Ok Tedi Deposit, Papua New Guinea: Trace Element Geochemistry and High-Precision Geochronology of Igneous Zircon. *Econ. Geol.* **113**, 39-61 (2018).
[23] Lang, J. R. et al. Geology and magmatic-hydrothermal evolution of the giant Pebble porphyry copper-gold-molybdenum deposit, Southwest Alaska. *Econ. Geol.* **108**, 437-462 (2013).
[24] Li, Y., Selby, D., Feely, M., Costanza, A. & Li, X.-H. Fluid inclusion characteristics and molybdenite Re-Os geochronology of the Qulong porphyry copper-molybdenum deposit, Tibet. *Min. Dep.* **52**, 137-158 (2017).
[25] Razique, A., Tosdal, R. M. & Creaser, R. A. Temporal evolution of the western porphyry Cu-Au systems at Reko Diq, Balochistan, western Pakistan. *Econ. Geol.* **109**, 2003-2021 (2014).
[26] Deckart, K., Clark, A. H., Cuadra, P. & Fanning, M. Refinement of the time-space evolution of the giant Mio-Pliocene Río Blanco-Los Bronces porphyry Cu-Mo cluster, Central Chile: New U-Pb (SHRIMP II) and Re-Os geochronology and $^{40}Ar/^{39}Ar$ thermochronology data. *Mineralium Deposita* **48**, 57–79 (2013).
[27] Annen, C., Blundy, J. D., & Sparks, R. S. J. The genesis of intermediate and silicic magmas in deep crustal hot zones. *J. Pet.* **47**, 505–539 (2006).
[28] Chiaradia, M. & Caricchi, L. Stochastic modelling of deep magmatic controls on porphyry copper deposit endowment. *Sci. Rep.* **7**, 44523 (2017).
[29] Newman, S. & Lowenstern, J. B. VolatileCalc: a silicate melt-H_2O-CO_2 solution model written in Visual Basic for Excel. *Computers and Geosciences* **28**, 597-604 (2002).
[30] Chiaradia, M. Copper enrichment in arc magmas controlled by over- riding plate thickness. *Nature Geoscience* **7**, 43–46 (2014).
[31] Le Bas, M.J., Le Maitre, R.W., Streckeisen, A. & Zanettin, B. A chemical classification of volcanic rocks based on the total alkali-silica diagram. *J. Pet.* **27**, 745-750 (1986).
[32] Plank, T., Kelley, K. A., Zimmer, M. M., Hauri, E. H. & Wallace, P. J. Why do mafic arc magmas contain ~4 wt% water on average? *Earth Planet. Sci. Lett.* **364**, 168-179 (2013).
[33] Simon, A. C. et al. Gold partitioning in melt–vapor–brine systems. *Geochim. Cosmochim. Acta* **69**, 3321-3335 (2005).
[34] Moss, R., Scott, S. D. & Binns, R. A. Gold Content of Eastern Manus Basin Volcanic Rocks: Implications for Enrichment in Associated Hydrothermal Precipitates. *Econ. Geol.* **96**, 91-107 (2001).
[35] Rock, N. M. S. & Groves, D. I. Do lamprophyres carry gold as well as diamonds? *Nature* **332**, 253-255 (1988).
[36] Georgatou, A. & Chiaradia, M. Magmatic sulphides in high-K calc-alkaline to shoshonitic and alkaline rocks. *Solid Earth*, https://doi.org/10.5194/se-2019-106 (2019).
[37] Annen, C. From plutons to magma chambers: Thermal constraints on the accumulation of eruptible silicic magma in the upper crust. *Earth Planet. Sci. Lett.* **284**, 409–416 (2009).
