# Supplementary material for: Gold endowments of porphyry deposits controlled by precipitation efficiency
Source: Nat Commun. 2020 Jan 14;11:248. doi: 10.1038/s41467-019-14113-1 (PMC6959242; doi:10.1038/s41467-019-14113-1)
Supplement: Supplementary file 3 — Description of Additional Supplementary Files [file 41467_2019_14113_MOESM3_ESM.pdf]

Description of Additional Supplementary Files

File Name: Supplementary Data 1

Description: Geological, geochemical, geochronological and endowment data of 118 porphyry copper deposits retrieved from the literature

File Name: Supplementary Data 2

Description: Monte Carlo simulations algorithm
